# The complete mitochondrial genome of the Thomas’s horseshoe bat (*Rhinolophus thomasi*) using next-generation sequencing and Sanger sequencing

**DOI:** 10.1080/23802359.2016.1266707

**Published:** 2016-12-23

**Authors:** Yutong Xing, Xiuguang Mao

**Affiliations:** Institute of Estuarine and Coastal Research, East China Normal University, Shanghai, China

**Keywords:** Bat, mitogenome, museum, *Rhinolophus thomasi*

## Abstract

The Thomas’s horseshoe bat (*Rhinolophus thomasi*) is restricted to southeast Asia and few studies have been conducted on this species. Here we characterized the complete mitochondrial genome of *R. thomasi* using next-generation sequencing and Sanger sequencing. The whole mitogenome is 16,899 bp in length and contains 22 tRNA, 2 rRNA, 13 protein-coding genes and a non-coding control region. The tissue used in this study was taken from a sample collected 13 years ago and the genomic DNA was seriously degraded. Our study adds to a growing number of studies indicating that next-generation sequencing is powerful in generating genetic/genomic resources from museum samples.

The Thomas’s horseshoe bat (*Rhinolophus thomasi*), belonging to Rhinolophidae, is distributed across the southeast Asia including Laos, Myanmar, Thailand and Vietnam (Csorba et al. 2003). The previous phylogenetic study suggested the occurrence of mitochondrial introgression between *R. thomasi* and its closely related species (*R. sinicus*) in Yunnan province, China (Mao et al. 2013). In this study we generated a complete mitogenome of *R. thomasi* (GenBank accession: KY124333) from outside of China which will help to understand the phylogenetic relationship between *R. thomasi* and it’s closely related species.

In this study muscle tissue was taken from a male adult *R. thomasi* sampled from Shan State, Myanmar in 2003 (HZM1.35115, the Harrison Institute, UK). Genomic DNA was extracted using DNeasy Kits (Tiangen, China). The DNA was seriously degraded and its concentration was extremely low. To obtain enough genomic DNA for library construction, we performed whole genome amplification (WGA) using WGA kit (Sigma-Aldrich) under the protocol provided in the kit. Then a DNA library with insert fragments of ∼350 bp was constructed using an Illumina TruSeq DNA sample preparation kit (Illumina Inc., San Diego, CA) and sequenced on an Illumina Hiseq4000 sequencer (150 bp paired-end). Raw reads were filtered by trimming unknown or low quality bases (Phred quality score <20) using NGSQCToolkit_v.2.3.3 (Dai et al. 2010) and only reads with the length of over 120 after trimming were retained.

After quality control, a total of 3,161,338 reads (964 Mb) were used for further analysis. We used two complementary methods to generate the mitogenome of *R. thomasi*. Firstly, filtered reads were *de novo* assembled into contigs using Trinity software (v2.2.0) (Haas et al. 2013) with default parameters. To identify mitochondrial DNA sequences of *R. thomasi*, we performed BLASTN searches on assembled contigs using the complete mitogenome of *R. sinicus sinicus* (GenBank accession: KR106992) as a query. We found a contig (7418 bp) containing nucleotide sequences of the mitogenome from 1758 to 9176 bp.

To complement the *de novo* assembly method, we mapped filtered reads onto the mitogenome of *R. sinicus sinicus* using BWA-0.5.7 (Li & Durbin 2009) with default parameters. A series of options in SAMtools (v0.1.19) (Li et al. 2009) were used to generate a consensus sequence. By combining the results from the above two methods, we generated a nearly complete mitochondrial sequence of *R. thomasi* (16,506 bp). The missing nucleotide sequences including parts of the *nd5* gene and D-loop were obtained using traditional Sanger sequencing. The *nd5* gene was amplified using two pairs of primers (Primer 1: forward primer, 5′-GGGGATGAGCAGGACA-3′ and backward primer, 5′-TTTGGGTAGGGCGTTT-3′; Primer 2; forward primer, 5′-CGCCTGAGCCCTTCTAAT-3′ and backward primer, 5′-CGGGTGGTCTTTACTTGTT-3′). The thermocycling profile for *nd5* was: 95 °C for 5 min; 34 cycles of 30 s at 94 °C, 30 s at 56 °C and 40 s at 72 °C; 72 °C for 10 min. PCR primers and thermocycling profile for D-loop have been described previously (Castella et al. 2001). Three different base calls were observed on *nd5* gene between Sanger sequencing and next-generation sequencing in a length of 780 bp. The average sequence depth of 38.64 was obtained by mapping reads to the final mitochondrial sequence.

The final mitogenome of *R. thomas*i is 16,899 bp long with a base composition of 14.31% G, 31.60% A, 25.01% T, and 29.08% C. It consists of 22 tRNA genes, 2 rRNAs genes, 13 protein-coding genes, and a non-coding control region (D-loop). Except for *nd6* and eight tRNA genes which are encoded on the L-strand, other genes are encoded in the H-strand. The total length of 13 protein-coding genes is 11,408 bp. All genes initiate with an ATG codon except for *nd2*, *nd3* and *nd5* with an ATA. Eight protein-coding genes terminate with a conventional stop codon (TAA or TAG) and *cytb* with AGA. An incomplete stop codon (TA- or T–) is observed in *nd1*, *nd2*, *nd4* and *cox3*. To verify the sequence of *R. thomasi*, we conducted a phylogenetic analysis on mitogenomes from 13 *Rhinolophus* bats, including *R. thomasi*, and one outgroup *Hipposideros armiger*. Sequences from 13 protein-coding genes were concatenated and aligned using MEGA7 (Kumar et al. 2016). Then, a maximum-likelihood (ML) tree was constructed using RAxML 7.2.8 (Berger et al. 2011) with GTRGAMMA model. Bootstrap supports were estimated from 1000 replicate searches. The ML tree supported the classification of *R. thomasi* with *R. sinicus* with 100% bootstrap probability (Figure 1), as shown in previous studies (e.g. Csorba et al. 2003; Mao et al. 2013).

**Figure 1.**
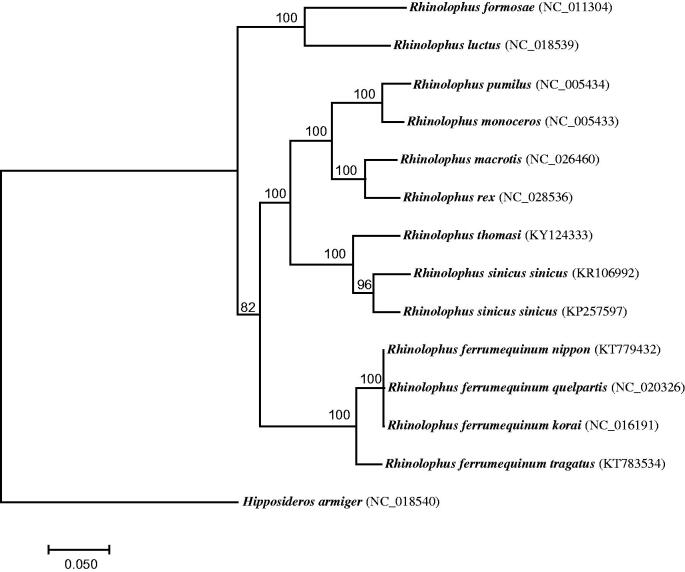
A maximum-likelihood tree reconstructed based on 14 bat mitogenomes. *Hipposideros armiger* was used as the outgroup. GenBank accession for each bat mitogenome was shown in bracket.
